# Antibiotic Prophylaxis and Treatment of Neonatal Group B Streptococcus Disease in the Era of Antimicrobial Resistance

**DOI:** 10.3390/antibiotics15030306

**Published:** 2026-03-18

**Authors:** Chryssoula Tzialla, Serena Salomè, Vito Mondì, Vincenzo Salvo, Alberto Berardi

**Affiliations:** 1 Neonatal Intensive Care Unit, ASST Papa Giovanni XXIII, 24127 Bergamo, Italy; 2 Division of Neonatology, Department of Translational Medical Sciences, University of Naples Federico II, 80138 Naples, Italy; serena.salome@unina.it; 3 Neonatology and Neonatal Intensive Care Unit, Azienda Ospedaliera San Giovanni-Addolorata, 00184 Rome, Italy; vmondi@hsangiovanni.roma.it; 4 Neonatal Intensive Care Unit, Ospedale “Giovanni Paolo II”, 97100 Ragusa, Italy; vincenzo.salvo@asp.rg.it; 5 Neonatal Intensive Care Unit, Women’s and Children’s Health Department, University Hospital of Modena, 41124 Modena, Italy; alberto.berardi@unimore.it

**Keywords:** Group B Streptococcus, neonatal sepsis, intrapartum antibiotic prophylaxis, antimicrobial resistance, penicillin G, vancomycin, clindamycin resistance

## Abstract

Group B Streptococcus (GBS) remains a major cause of early- and late-onset neonatal sepsis worldwide, despite the widespread use of intrapartum antibiotic prophylaxis (IAP). β-lactam antibiotics, including penicillin G and ampicillin, remain the cornerstone of both GBS prophylaxis and neonatal treatment, supported by sustained susceptibility, favorable pharmacokinetics, and extensive clinical experience. However, increasing global resistance to macrolides and lincosamides has markedly reduced the reliability of clindamycin and erythromycin, which are commonly used as second-line agents in women with severe penicillin allergy. This narrative review summarizes current evidence on antibiotic strategies for the prevention and treatment of neonatal GBS disease, with a particular focus on antimicrobial resistance patterns and their clinical implications. Available surveillance data demonstrate substantial geographic variability in resistance but consistently low resistance to β-lactams and vancomycin. These trends have expanded the role of vancomycin in IAP for women with high-risk β-lactam allergy and in neonatal treatment when first-line agents are contraindicated. Alternative agents such as linezolid and teicoplanin exhibit activity against GBS, but their use remains limited by sparse neonatal data and pharmacokinetic variability. Ongoing antimicrobial surveillance, susceptibility-guided therapy, and stewardship initiatives are essential to preserve effective GBS prevention and treatment strategies.

## 1. Introduction

Group B Streptococcus (GBS) disease remains a leading cause of neonatal morbidity and mortality worldwide, particularly during the first three months of life [1,2,3,4]. Neonatal GBS disease is classically categorized into early-onset disease (EOD) and late-onset disease (LOD), both of which are associated with severe clinical manifestations, including sepsis, pneumonia, and meningitis [1,2,3,4]. Invasive GBS infection contributes substantially to neonatal and early childhood mortality and is frequently associated with long-term neurodevelopmental sequelae secondary to brain injury [1,2].

Recent global estimates indicate that, in 2020, EOD and LOD collectively affected approximately 400,000 neonates and young infants, resulting in nearly 60,000 infant deaths worldwide. Mortality exceeded 90,000 deaths in settings lacking access to skilled birth attendants, and an estimated 37,000 surviving children developed lifelong disabilities attributable to invasive GBS disease [5]. These figures underscore the persistent and disproportionate global burden of GBS disease, particularly in low-resource settings.

This narrative review summarizes the epidemiology of neonatal infection, and current evidence on antibiotic strategies for the prevention and treatment of neonatal GBS disease, with a particular focus on antimicrobial resistance patterns and their clinical implications.

## 2. Neonatal GBS Disease

### 2.1. Early-Onset Disease

Early-onset disease is defined by the isolation of GBS from normally sterile sites, such as blood or cerebrospinal fluid, within the first 6 days of life [3,4]. However, contemporary surveillance data indicate that approximately 95% of EOD cases occur during the birth hospitalization, most frequently within the first 12–48 h after delivery [3,4,6]. Transmission primarily occurs via vertical exposure from colonized mothers during labor or delivery [1,4,6].

The global incidence of EOD varies substantially by region, with the highest burden observed in Africa (approximately 90,800 cases annually) and the lowest incidence reported in Europe and North America (approximately 4300 cases) [5].

Rectovaginal GBS colonization affects an estimated 11–35% of pregnant women worldwide [1,4,7], corresponding to nearly 20 million colonized women in 2020 alone [5,8]. Consequently, the prevention of maternal colonization and interruption of vertical transmission constitute key priorities in perinatal healthcare strategies. Current prevention efforts rely on intrapartum antibiotic prophylaxis (IAP), which is associated with a marked reduction in EOD risk [4,6,7,9]. IAP implementation varies by region and includes risk factor-based strategies, universal microbiology-based screening, and hybrid approaches [7,8,10,11]. In risk-based strategies, IAP is administered when predefined maternal or obstetric risk factors are present, whereas universal screening strategies involve the antenatal testing of all pregnant women for GBS colonization, with IAP reserved for those testing positive [7,10].

A recent meta-analysis of observational studies demonstrated that microbiology-based screening strategies were associated with an approximately 70% reduction in EOD risk compared with no preventive policy and a 60% reduction compared with risk factor-based approaches [11]. Specifically, the relative risk of EOD after the implementation of screening-based strategies was 0.37 (95% CI, 0.25–0.55), whereas the relative risk following risk factor-based strategies was 0.65 (95% CI, 0.48–0.87) [11]. Since the introduction of IAP, the incidence of EOD has declined by more than 80% in high-income countries [4,6,7]. Nevertheless, the global incidence of EOD remains approximately 0.41 cases per 1000 live births, with case fatality rates ranging from 4% to 10% in high-income settings and substantially higher mortality reported in low-income regions [1,3,5].

Although preterm infants—particularly those born between 22 and 28 weeks’ gestation—experience a significantly higher individual risk of EOD, approximately 75% of cases occur in term infants, among whom GBS remains a leading cause of early-onset sepsis [1,3].

### 2.2. Late-Onset Disease

Late-onset disease occurs between 7 and 89 days of life and may result from vertical transmission, nosocomial exposure, or community acquisition [1,3,4]. Unlike EOD, in the United States, the incidence of LOD has remained relatively stable over the past two decades, with global estimates ranging from 0.3 to 0.4 cases per 1000 live births [3,4,12]. Notably, epidemiological data from the United Kingdom and the Netherlands suggest a trend of rising LOD rates [13,14].

LOD has emerged as the predominant form of neonatal GBS disease in several high-income countries, accounting for approximately 57% of all reported neonatal GBS cases in the United States in 2020 [3]. Consistent with this shift, French surveillance data demonstrate a 65% increase in LOD incidence over the past two decades [15]. Preterm infants are at particularly high risk, with an estimated sixfold greater likelihood of developing LOD compared with term infants [3].

Importantly, the incidence of LOD has not been reduced by IAP implementation, and no effective preventive strategies currently exist for late-onset neonatal GBS disease [3,4,16]. This limitation highlights the urgent need for alternative preventive approaches, including maternal vaccination.

## 3. Antibiotics for Prophylaxis and Treatment of GBS Infections

β-lactam antibiotics remain the cornerstone of both intrapartum prophylaxis and treatment of GBS infections in neonates [4,7].

### 3.1. Treatment of Neonatal GBS Infection

Penicillin G is the recommended first-line agent for confirmed neonatal GBS infection, with ampicillin considered an acceptable alternative [4,7]. Based on prospective EOS surveillance data, clinical GBS isolates remain almost universally susceptible to β-lactam antibiotics, supporting penicillin G as the preferred agent given its narrow antimicrobial spectrum, excellent safety profile, and proven clinical efficacy [4,17].

Pharmacokinetic and pharmacodynamic studies in neonatal populations support current dosing recommendations for penicillin G. Administering penicillin G at 50,000 IU/kg every 12 h for EOD achieves therapeutic serum concentrations in both term and preterm neonates. Dose adjustments based on gestational age and postnatal age are recommended to optimize target exposure while minimizing the risk of drug accumulation and toxicity, particularly in extremely preterm infants with immature renal clearance [18].

Despite its broader antimicrobial spectrum, ampicillin remains a valid and acceptable alternative for the treatment of GBS infections, since it is effective for the treatment of neonatal GBS infections, including bacteremia and meningitis. The recommended dosing for bacteremia ranges from 50 to 100 mg/kg every 8–12 h, with the higher dose required for meningitis to ensure adequate cerebrospinal fluid penetration [18,19].

Empiric antibiotic therapy for suspected EOD typically includes a combination of ampicillin and gentamicin, providing synergistic bactericidal activity against GBS while also covering other common neonatal pathogens [20]. Once blood or cerebrospinal fluid cultures confirm GBS as the causative organism, antimicrobial therapy should be streamlined to penicillin G or ampicillin monotherapy in accordance with antimicrobial stewardship principles (Table 1) [4,20].

The duration of antimicrobial therapy is guided by the site and severity of infection. According to the American Academy of Pediatrics (AAP), a 10-day course of intravenous antimicrobial therapy is generally recommended for uncomplicated GBS bacteremia, whereas meningitis requires a longer treatment duration of 14–21 days [4]. In contrast, the NICE guidelines recommend a 7-day course of antibiotics for neonates with sepsis without meningitis, provided that the infant has achieved full clinical recovery [21]. Notably, neither the AAP nor NICE recommendations are strictly evidence-based, and high-quality evidence is lacking. Available randomized clinical trials related to the duration of antibiotic therapy suggest that a 10-day course for confirmed, uncomplicated neonatal septicemia is as effective as a 14-day regimen, provided that clinical remission is achieved by day 7 of treatment (Table 2) [22,23,24,25,26]. Furthermore, two randomized controlled trials involving neonates with culture-positive sepsis demonstrated that a 7-day course of intravenous antibiotics may not be inferior to a 10-day or 14-day course in carefully selected infants with uncomplicated culture-proven neonatal sepsis who achieved clinical stability within 5 days of therapy, have sterile follow-up cultures, and have no meningitis or focal infection (Table 2) [27,28,29]. However, several important limitations should be acknowledged when interpreting these findings. Many of the available trials were conducted in low- and middle-income settings, which may differ substantially from high-resource settings in terms of patient populations, pathogen profiles, and antibiotic sensitivity patterns. In fact, in high-income countries, GBS continues to be a leading cause of early-onset sepsis, although an increasing proportion of cases are associated with antibiotic-resistant *Escherichia coli* [30]. In contrast, coagulase-negative staphylococci (CONS) and *Staphylococcus aureus* are the most common pathogens responsible for late-onset sepsis [31]. In low- and middle-income settings, the distribution of pathogens differs, with *Klebsiella* (26%), *Staphylococcus aureus* (23%), CONS (23%), and *E. coli* (15%) being most frequently identified, and with similar pathogen profiles observed in both early- and late-onset sepsis [32]. In addition, pathogen-specific subgroup analyses focusing exclusively on GBS are limited, making it difficult to draw firm conclusions specifically for this organism. Consequently, the generalizability of these results—particularly to high-resource NICU settings and to cases of GBS sepsis—remains uncertain and warrants cautious interpretation.

With regard to the duration of antibiotic therapy for bacterial meningitis in infants younger than 3 months, current recommendations are largely based on expert consensus. A recent systematic review of the literature found that the available evidence, which relies predominantly on observational studies with small sample sizes, is insufficient to support current treatment recommendations or to define the optimal duration of parenteral antibiotic therapy [33]. Throughout therapy, close clinical monitoring and assessment of laboratory markers of infection are essential to ensure therapeutic response in confirmed sepsis [4,20,34].

### 3.2. Antibiotic Stewardship in Neonatal GBS Management

Antibiotic stewardship plays a central role in the management of neonatal GBS infection and should be integrated into everyday clinical decision-making. Once GBS is identified as the causative pathogen, therapy should be promptly narrowed to a targeted β-lactam regimen, avoiding unnecessarily broad-spectrum coverage. Equally important is the avoidance of prolonged antibiotic courses without clear indication, particularly in cases of culture-negative sepsis. Empiric antibiotics should be discontinued early—typically within 36–48 h—when blood cultures remain negative and the infant’s clinical condition is reassuring.

When clinically appropriate, shortening the duration of therapy may provide meaningful benefits. These include reducing the risk of antimicrobial resistance, limiting disruption to the developing neonatal microbiota, decreasing complications associated with invasive vascular access, and potentially shortening the length of stay in hospital.

Nevertheless, stewardship efforts must never compromise patient safety. In cases of confirmed meningitis or complicated infection, prolonged and appropriately dosed therapy remains essential, and any attempt to abbreviate treatment should be avoided in the absence of robust supporting evidence.

### 3.3. Intrapartum Antibiotic Prophylaxis

In the context of IAP, intravenous penicillin G is the preferred agent due to its proven efficacy, favorable maternal and neonatal safety, and excellent placental transfer [7]. Intravenous ampicillin is an acceptable alternative when penicillin G is unavailable (Table 2) [7,35,36].

For pregnant women with a documented penicillin allergy, antibiotic selection for IAP is guided by the severity of the allergic reaction and local GBS susceptibility patterns. For individuals with a low-risk penicillin allergy (i.e., a history not consistent with IgE-mediated hypersensitivity), cefazolin is recommended by the American College of Obstetricians and Gynecologists (ACOG) (Table 3) [7]. Cefazolin exhibits rapid and effective transplacental transfer, with cord blood and amniotic fluid concentrations exceeding the minimum inhibitory concentration (MIC) for GBS within approximately 20 min of administration, supporting its efficacy in preventing vertical transmission [7,36,37].

In women with a high-risk penicillin allergy, such as presumed IgE-mediated hypersensitivity reactions, clindamycin may be considered. However, given the increasing prevalence of clindamycin resistance and inducible resistance (D-zone test positivity), as well as its less reliable placental transfer, clindamycin should be used only when the GBS isolate is confirmed to be susceptible and when no safer alternatives are available. In all other cases, vancomycin is the recommended and validated alternative for IAP, owing to its reliable transplacental passage and sustained activity against nearly all GBS isolates (Table 3) [4,7,10,36,37].

The majority of women who report a penicillin allergy are actually able to tolerate the drug. Therefore, penicillin allergy testing should be promoted in obstetric care, as it is safe during pregnancy and advantageous for all patients with a reported allergy—especially when the clinical history suggests a possible IgE-mediated reaction, an unclear level of severity, or both. Confirming the absence of a type I hypersensitivity reaction removes the need to rely on alternatives antibiotics to penicillin for IAP against GBS EOD.

Collectively, these recommendations are supported by pharmacokinetic and pharmacodynamic data demonstrating effective fetal and neonatal exposure, clinical efficacy in preventing EOD, and the necessity of avoiding antimicrobial agents with high resistance rates or suboptimal placental transfer. Careful adherence to guideline-based antibiotic selection remains essential to maximize prophylactic and therapeutic effectiveness while supporting antimicrobial stewardship [7,37].

## 4. Antimicrobial Resistance Patterns and Global Trends in GBS

Antimicrobial resistance patterns among GBS isolates exhibit marked variability across geographic regions, study populations, and time periods, complicating efforts to define uniform global susceptibility profiles. A comprehensive systematic review encompassing 266 studies from 57 countries highlighted substantial heterogeneity in reported resistance rates, reflecting differences in regional antibiotic use, circulating GBS lineages, surveillance methodologies, and laboratory testing practices [38].

Despite this variability, GBS continues to show consistent susceptibility to β-lactam antibiotics, including penicillin, ampicillin, and cefazolin, which continue to represent first-line agents for both neonatal GBS infection and IAP [1,3,4,7,12,39]. Recent multicenter surveillance studies and whole-genome sequencing analyses confirm that penicillin resistance in GBS is exceedingly rare, with no documented clinical treatment failures attributable to β-lactam resistance in neonates to date [12,16]. These findings reinforce the continued reliability of β-lactam antibiotics as the foundation of GBS prevention and treatment strategies.

A systematic review of 169 studies involving pregnant women from diverse geographic regions reported pooled resistance rates of 0.7% for penicillin, 1.0% for ampicillin, 1.34% for cefazolin, and 0.59% for vancomycin, with the highest rates observed in Africa (penicillin 6%, ampicillin 4%, vancomycin 2%) [40]. Similarly, a global meta-analysis of invasive GBS infections reported low rates of resistance to ampicillin (1.7%), penicillin (3.1%), and vancomycin (1.4%) [38]. Importantly, these low resistance levels have not demonstrated a consistent upward temporal trend, supporting the continued use of these agents as first-line therapies.

In contrast, resistance to macrolides and lincosamides—specifically erythromycin and clindamycin—has increased substantially worldwide. These agents are commonly used as second-line options for IAP in women with severe penicillin allergy, rendering rising resistance rates of significant clinical concern [7,41]. Surveillance data consistently demonstrate pronounced geographic variability and a progressive increase in resistance over the past two decades [12,38,42]. A global meta-analysis of invasive GBS isolates reported resistance rates of 29.3% for clindamycin and 35.0% for erythromycin, with the highest prevalence observed in North America and parts of Europe, and lower but steadily increasing rates reported in Eastern Europe and Asia [38].

Among pregnant women, pooled resistance rates are similarly high. A large systematic review reported resistance rates of 20.0% for clindamycin (140 studies) and 21.5% for erythromycin (159 studies), with Asia exhibiting the highest continental resistance rates (28% and 32%, respectively) [40]. These findings underscore the limited reliability of macrolides and lincosamides for empirical use in IAP without prior susceptibility testing.

Data from the United States illustrate both high resistance rates and pronounced regional variation. Multicenter surveillance of invasive GBS isolates from infants and adults reported clindamycin resistance rates ranging from 50.9% to 55.4% and erythromycin resistance rates from 61.7% to 75.5%, particularly among serotypes V and II [42]. National surveillance conducted between 2006 and 2016 documented a significant increase in erythromycin resistance (from 34.7% to 49.1%) and clindamycin resistance (from 14.7% to 26.0%), with inducible clindamycin resistance accounting for an additional 7% of isolates [43,44].

Outside the United States, resistance patterns show similarly concerning trends. In New Zealand, approximately one-third of early-onset neonatal GBS isolates demonstrate resistance to erythromycin and/or clindamycin [45]. European surveillance studies report elevated resistance rates, particularly among serotype V and the hypervirulent ST-17 clone [2,46]. In Italy, a recent single-center study documented erythromycin resistance rates of 33.5% and clindamycin resistance rates of 29.5% among maternal colonization isolates, rising to 57.1% among early-onset neonatal GBS isolates. These findings were largely attributed to the expansion of a multidrug-resistant ST-17 subclone of serotype III and exceeded resistance levels reported in earlier studies from 2015 to 2019 [46,47,48].

In Central and Northern Europe, resistance trends appear more heterogeneous but are increasing. Polish surveillance data from 2024 reported macrolide and lincosamide resistance in 7.9% of GBS isolates from pregnant women and neonates, predominantly associated with ermB and serotype V, with an upward trend noted [49]. Longitudinal data from Norway (2004–2018) demonstrated a gradual but statistically significant increase in erythromycin and clindamycin resistance, driven primarily by horizontal gene transfer via mobile genetic elements rather than clonal expansion [50]. In France, erythromycin resistance among invasive infant GBS isolates increased from 22% to 30% over a 13-year surveillance period [15].

Data from Asia indicate particularly high resistance rates. Surveillance data from the China Antimicrobial Resistance Surveillance System (2017–2021) reported very high resistance to erythromycin and clindamycin, with a modest decline observed between 2018 and 2020 followed by a renewed increase in 2021 [51].

Extremely high resistance rates have been consistently reported for tetracyclines, with global resistance approaching 80%, as well as for doxycycline (64.9%), azithromycin (41.0%), and clarithromycin (43.4%) [12,15,38,39,46,50]. Although these agents are not recommended for neonatal GBS treatment or intrapartum prophylaxis, their high resistance rates reflect widespread selective pressure and horizontal gene transfer within GBS populations. In contrast, resistance remains rare for meropenem (0.7%), daptomycin (0.3%), and linezolid (0.8%) [38]. Reduced susceptibility to aminoglycosides (gentamicin, amikacin) and fluoroquinolones has been reported but remains uncommon in most regions and has limited clinical relevance in neonatal practice [15,38,39].

The global resistance estimates reported in the literature should be interpreted with caution due to substantial heterogeneity across regions, study designs, and laboratory methodologies. Resistance rates may vary significantly between geographic areas, reflecting differences in antimicrobial use, local epidemiology, and surveillance systems. In addition, variations in study design (e.g., retrospective versus prospective studies, single-center versus multicenter cohorts) and laboratory methods for antimicrobial susceptibility testing, including the use of different interpretative criteria (e.g., CLSI versus EUCAST breakpoints), may further limit direct comparability between studies.

Overall, these data highlight the growing challenge posed by macrolide and lincosamide resistance in GBS and emphasize the importance of continued antimicrobial susceptibility surveillance to inform clinical guidelines, optimize intrapartum prophylaxis, and support antimicrobial stewardship efforts worldwide.

## 5. Antibiotics for Prophylaxis and Therapy of Resistant GBS Strains

### 5.1. Intrapartum Antibiotic Prophylaxis for Resistant Strains

The increasing resistance to clindamycin and erythromycin among GBS isolates has significant clinical implications, particularly for IAP in women with documented penicillin allergy. Elevated resistance rates substantially reduce the effectiveness of these agents and are associated with a higher risk of prophylaxis failure when clindamycin or erythromycin is administered empirically without prior susceptibility testing. Clinical consequences include increased maternal infectious morbidity and persistent maternal GBS colonization at the time of delivery, thereby increasing the risk of neonatal exposure [41].

Evidence from a recent cohort study indicates that women receiving clindamycin for IAP in the absence of confirmed susceptibility experience significantly higher rates of intrapartum fever, clinical chorioamnionitis, postpartum fever, and postpartum antibiotic use compared with those receiving β-lactam agents such as ampicillin. In addition, GBS is more frequently isolated from chorioamniotic cultures following clindamycin prophylaxis, suggesting reduced intra-amniotic activity and inadequate suppression of GBS during labor [41]. Collectively, these findings underscore the clinical risks associated with reliance on second-line agents in the setting of unrecognized or inducible resistance.

In this context, vancomycin is recommended as the preferred alternative agent for IAP in women with severe penicillin allergy when resistance to clindamycin or erythromycin is known or suspected (Table 2). Guidelines from the AAP, ACOG, and BJOG support the use of vancomycin in such cases, given its consistent activity against GBS despite rare reports of non-vancomycin-susceptible isolates [4,7,10,44,52]. Clinical and pharmacokinetic studies further strengthen the evidence base for intrapartum vancomycin use, demonstrating adequate maternal–fetal transfer, safety, and efficacy and favorable neonatal outcomes [37,53,54,55]. Weight-based maternal dosing achieves umbilical cord blood concentrations exceeding the GBS minimum inhibitory concentration in more than 90% of neonates, supporting its microbiological efficacy in preventing EOD [55].

Observational studies involving large obstetric cohorts have documented a steady increase in vancomycin use for GBS prophylaxis over the past decade, reflecting its expanding role in response to rising clindamycin and erythromycin resistance [41,55,56]. Importantly, neonatal outcomes following maternal vancomycin exposure are comparable to those observed with β-lactam prophylaxis, with no increase in EOD, neonatal intensive care unit admission, neonatal bacteremia, or length of hospitalization [37]. Although neonates exposed to vancomycin undergo postnatal laboratory evaluation more frequently, this appears to reflect heightened clinical vigilance rather than drug-related toxicity [37]. Available safety data indicate that vancomycin is generally well tolerated in pregnant individuals, with infrequent maternal adverse events and no evidence of increased neonatal toxicity or renal dysfunction when appropriate dosing and monitoring are applied [55,57]. Nonetheless, antimicrobial stewardship remains critical, as unnecessary vancomycin use may contribute to the emergence of vancomycin-resistant organisms, particularly *Enterococcus* spp., with important public health implications [6,7].

### 5.2. Therapeutic Options for Resistant Strains in Neonates

To date, no cases of neonatal GBS infections caused by β-lactam-resistant strains have been reported [4,38,44,52]. Therefore, although alternative molecules to β-lactams are mentioned as potential therapeutic options for neonatal GBS infections, their inclusion should be regarded as purely informative rather than reflective of current clinical necessity. These alternative agents are indeed realistic options, as they are already used in neonates for the treatment of infections caused by resistant Gram-positive organisms. However, in the specific context of neonatal GBS disease, β-lactams remain fully effective. Consequently, the discussion of non-β-lactam therapies in the article is intended to provide comprehensive information rather than to suggest an immediate or practical need for their use in clinical practice.

Vancomycin is also the recommended alternative for the treatment of neonatal GBS infection when resistance to β-lactam antibiotics is suspected or confirmed, as it retains excellent activity against GBS. Vancomycin resistance among neonatal GBS isolates remains exceedingly rare. Large-scale U.S. surveillance conducted between 2015 and 2017 identified only two non-vancomycin-susceptible isolates among 6340 invasive GBS cases, both harboring the vanG gene and exhibiting a MIC of 2 µg/mL [58]. Similarly, a 2024 global meta-analysis reported extremely low resistance rates with no evidence of increasing temporal trends [38]. Both historical and contemporary studies consistently demonstrate near-universal susceptibility of neonatal invasive GBS isolates to vancomycin [15,59,60].

A pharmacokinetic study in neonatal populations suggests that vancomycin trough concentrations of approximately 6.8–11 µg/mL and a 24 h area under the concentration–time curve (AUC_(24)_) of around 240 mg·h/L are associated with clinical and microbiological cure [61]. However, vancomycin therapy carries a recognized risk of nephrotoxicity and, rarely, ototoxicity, particularly at elevated trough concentrations or when administered concomitantly with other nephrotoxic agents [62]. These risks underscore the importance of therapeutic drug monitoring, especially in preterm neonates and those receiving prolonged therapy [46,47,48,49].

Several alternative antimicrobial agents have been investigated for the treatment of resistant Gram-positive infections in neonates. Linezolid, an oxazolidinone antibiotic, has demonstrated efficacy comparable to that of vancomycin and a favorable short-term safety profile in neonates with resistant Gram-positive infections, including GBS [63]. In a randomized clinical trial, linezolid achieved similar rates of clinical cure and pathogen eradication with fewer drug-related adverse events, supporting its use in selected cases of vancomycin intolerance or treatment failure [64]. Although long-term adverse effects specific to neonates have not been reported, hematologic monitoring is recommended during linezolid therapy—particularly for treatment durations exceeding 7–14 days—due to the risk of thrombocytopenia and transient leukopenia [65,66,67,68,69]. These hematologic effects are typically reversible upon drug discontinuation and rarely necessitate the early termination of therapy [70]. Given its cost and potential for toxicity with prolonged use, linezolid should be reserved for carefully selected cases of resistant Gram-positive infections.

Emerging agents such as tedizolid, a next-generation oxazolidinone, exhibit excellent in vitro activity against GBS, with extremely low global resistance rates (~0.1%) [38]. However, clinical data in neonates are currently lacking, and tedizolid is not recommended for GBS prophylaxis or treatment in this population [63]. Although a phase 3 trial has demonstrated safety and efficacy in pediatric patients aged 28 days to <12 years with acute bacterial skin and skin structure infections, neonatal-specific data remain unavailable [71].

Teicoplanin, a glycopeptide antibiotic with activity against GBS, is widely used in pediatric populations for the treatment of severe Gram-positive infections and is often considered an alternative to vancomycin due to its potentially more favorable adverse-effect profile, but its use in neonates is less established and not widely adopted in clinical practice. Nevertheless, recent systematic reviews highlight substantial interindividual variability in teicoplanin pharmacokinetics among neonates, and currently recommended dosing regimens frequently fail to achieve therapeutic target concentrations. These findings emphasize the need for individualized dosing strategies in neonatal populations, with careful consideration of gestational age, postnatal age, body weight, and renal function [72,73,74].

Third-generation cephalosporins, such as cefotaxime, are generally well tolerated in neonates and retain activity against GBS. However, they are not preferred for the treatment of resistant GBS strains due to concerns regarding the selection of resistant organisms and inferior efficacy compared with β-lactam antibiotics and glycopeptides in this context.

Other agents, including daptomycin, tigecycline, and ceftaroline, show consistent susceptibility patterns in surveillance studies, but are not the standard of care for neonatal GBS infection [38].

At present, no major clinical guidelines recommend the routine use of novel antimicrobial agents for GBS prophylaxis or treatment in neonates. Ongoing research efforts are largely focused on maternal vaccination strategies rather than the development of alternative antibiotics. Although several agents in the antimicrobial pipeline demonstrate activity against multidrug-resistant Gram-positive pathogens, none are currently approved or specifically recommended for the prevention or treatment of neonatal GBS disease [75,76,77].

## 6. Conclusions

Neonatal GBS disease continues to represent a significant global cause of morbidity and mortality. Although the widespread implementation of intrapartum antibiotic prophylaxis has substantially reduced early-onset disease, GBS infection remains a major clinical concern worldwide.

β-lactam antibiotics continue to be highly effective against GBS, with sustained universal susceptibility reported globally. This reassuring microbiological stability supports the continued use of penicillin as the first-line agent for both prophylaxis and treatment. In contrast, increasing resistance to macrolides and clindamycin has been observed in many regions, limiting their empirical use in penicillin-allergic women unless antimicrobial susceptibility is confirmed. Vancomycin remains a reliable alternative for intrapartum prophylaxis in women with high-risk penicillin allergy; however, its use should be carefully considered within antimicrobial stewardship frameworks to avoid unnecessary selection pressure and the emergence of resistant organisms.

Emerging evidence suggests that shortened antibiotic courses (7–10 days) may be safe in carefully selected neonates with uncomplicated GBS bacteremia who demonstrate rapid clinical improvement and microbiological clearance. Nevertheless, GBS meningitis continues to warrant prolonged therapy, as robust data supporting shorter regimens in this setting are lacking. Clinical judgment and careful patient selection therefore remain central when considering treatment duration.

Antibiotic stewardship in neonatal care requires a delicate balance: reducing unnecessary antibiotic exposure while ensuring that invasive infections are adequately treated. Avoiding both overtreatment and undertreatment is essential to optimize neonatal outcomes.

Future efforts should focus on strengthening the global surveillance of antimicrobial resistance, implementing standardized neonatal antimicrobial stewardship protocols, and conducting high-quality randomized trials to better define optimal treatment durations. In parallel, continued progress toward the development and implementation of effective maternal GBS vaccines represents a critical strategy for long-term disease prevention.

Overall, a structured, evidence-informed, and stewardship-driven approach is essential to improve clinical outcomes while minimizing the risk of antimicrobial resistance in neonatal GBS disease.

## Figures and Tables

**Table 1:** Practical management summary for neonatal GBS infection.

Clinical Scenario	First-Line Therapy	Alternative	Suggested Duration	Comments
Suspected EOD (empiric)	Ampicillin + Gentamicin	N/A	Pending cultures (36–48 h)	De-escalate once pathogen identified
Confirmed GBS Bacteremia (uncomplicated)	Penicillin G 50,000 UI/kg every 12 h (PNA ≤ 7 d) 8 h (PNA > 7 d)	Ampicillin *GA ≤ 34* w 50 mg/kg every 12 h (PNA ≤ 7 d) 75 mg/kg every 12 h (PNA > 7 d) *GA > 34* w 50 mg/kg every 8 h	10 days (consider 7 in selected stable cases)	Consider shorter course only if rapid clinical response
GBS meningitis	Penicillin G 150,000 UI/kg every 8 h (PNA ≤ 7 d) 125,000 UI/kg every 6 h (PNA > 7 d)	Ampicillin 100 mg/kg every 8 h (PNA ≤ 7 d) 75 mg/kg every 6 h (PNA > 7 d)	14–21 days	No strong evidence for shortening

N/A, not applicable; GA, gestational age; PNA, postnatal age.

**Table 2:** Summary of randomized controlled studies on the duration of antibiotic therapy.

	Study	Enrolment	Randomization criteria	Primary outcome	Comment
Gathwala G 2010 [22]	10 d vs. 14 d RCT	>32 wks, >1.5 kg Blood culture positive, no meningitis 60 neonates: 2 groups of 30 patients each	Clinical remission at day 7 and negative CRP	Treatment failure at 28 d: one treatment failure in each group	Both failure cases might have been fresh episodes of sepsis
Salama EI 2020 [23]	10 d vs. 14 d	>36 wks, >2 kg Blood culture positive, no meningitis and other deep-seated focal infections 30 neonates: 2 groups of 15 patients each	Clinical remission at conclusion of therapy	Treatment failure within 4 weeks: SCT 2 vs. LCT 1 *p* = 0.54	All failure cases were CRP-positive at the end of therapy
Reddy A 2022 [24]	10 d vs. 14 d pilot randomized RCT	>32 wks, >1.5 kg Blood culture positive, no meningitis and other deep-seated focal infections, no *S. aureus* infection 70 neonates: 2 groups of 35 patients each	Clinical remission with blood culture and sepsis screening negative on day 7 of appropriate antibiotic therapy	Treatment failure at 28 d SCT 1 vs. LCT 0 *p* = 0.386	Did not include neonates with *S. aureus* sepsis; hence, the findings cannot be generalized to all the culture-positive sepsis
Fursule A 2022 [25]	10 d vs. 14 d parallel open-label, noninferiority RCT	>1.5 kg Blood culture positive for GNB 113 neonates: SCT 58 vs. LCT 55	Clinical remission and negative blood culture after 7 days of appropriate antibiotic therapy	No treatment failure in either group	Focused only on neonates with GN sepsis, limiting the generalizability of the findings
Islam K 2023 [26]	10 d vs. 14 d RCT	234 neonates with blood culture positive (no deep-seated infections): 2 groups of 117 patients each	Clinical remission on day 9 of antibiotic therapy	Treatment failure at 30 d SCT 3.8% vs. LCT 1.7% *p* = 0.40	The primary outcome was defined as clinical sepsis, assessed by unblinded pediatricians, leading to a high risk of measurement bias
Rohatgi S 2017 [29]	7 d vs. 10 d RCT	>32 wks, >1.5 kg Blood culture positive, no meningitis and other deep-seated focal infections 132 neonates: 2 groups of 66 patients each	5 days of appropriate antibiotic therapy completed and clinical remission	Treatment failure at 28 d one treatment failure in each group	Both failure cases had sterile blood and cerebrospinal fluid cultures and reactive CRP
Chowdhary G 2006 [27]	7 d vs. 14 d single-blinded, RCT	>32 wks, >1.5 kg Blood culture positive, no meningitis and other deep-seated focal infections 69 neonates: SCT 34 vs. LCT 35	Clinical remission on day 5 of antibiotic therapy	Treatment failure SCT 5 vs. LCT 1, *p* = 0.19 *S. aureus* sepsis SCT 4 vs. LCT 0, *p* = 0.022 Non *S. aureus* sepsis 3.8% failure in both	Neonates with *S. aureus* sepsis require 14 days of antibiotics
Dutta S 2025 [28]	7 d vs. 14 d Multicenter non-inferiority RCT with masked outcome assessment	>1.0 kg Blood culture positive, no *S. aureus* and fungal infections, no deep-seated focal infections 261 neonates: SCT 126 vs. LCT 135	Clinical remission by day 5 of appropriate antibiotic therapy and sustained remission until day 7	Risk difference for relapse (definite or probable) by day 21 post-antibiotic completion −3.0%	7 d course of antibiotics is non-inferior to a 14 d course among neonates with culture-proven sepsis in clinical remission within 5–7 days of starting appropriate antibiotics

RCT = randomized controlled trial; SCT = short-course therapy; LCT = long-course therapy; CRP = C-reactive protein; GNB = Gram-negative bacteria; GPB = Gram-positive bacteria; EOS = early-onset sepsis; LOS = late-onset sepsis.

**Table 3:** Practical management summary for intrapartum antibiotic prophylaxis (IAP).

Clinical Scenario	First-Line Therapy	Alternative	Suggested Duration	Comments
IAP (no allergy)	Penicillin G	Ampicillin	During labor	Most effective strategy
IAP (low-risk allergy)	Cefazolin	N/A	During labor	Good placental transfer
IAP (high-risk allergy + susceptible isolate)	Clindamycin	N/A	During labor	Use only if susceptibility confirmed
IAP (high-risk allergy + resistant/unknown)	Vancomycin	N/A	During labor	Avoid overuse; stewardship essential

N/A not applicable.

## Data Availability

No new data were created or analyzed in this study. Data sharing is not applicable to this article.

## Abbreviations

The following abbreviations are used in this manuscript:

GBS	Group B Streptococcus
EOD	Early-onset disease
LOD	Late-onset disease
IAP	Intrapartum antibiotic prophylaxis
AAP	American Academy of Pediatrics
CONS	Coagulase-negative staphylococci
RCT	Randomized controlled trial
SCT	Short-course therapy
LCT	Long-course therapy
GNB	Gram-negative bacteria
GPB	Gram-positive bacteria
CRP	C-reactive protein
GA	Gestational age
PNA	Postnatal age
ACOG	American College of Obstetricians and Gynecologists
MIC	Minimal inhibitory concentration
